# Alzheimer's disease in Down syndrome

**DOI:** 10.1002/alz70856_096000

**Published:** 2025-12-23

**Authors:** Juan Fortea

**Affiliations:** ^1^ Sant Pau Memory Unit, Hospital de la Santa Creu i Sant Pau, Institut de Recerca Sant Pau ‐ Universitat Autònoma de Barcelona, Barcelona, Spain; Center for Biomedical Investigation Network for Neurodegenerative Diseases (CIBERNED), Madrid, Madrid, Spain; Catalan Foundation for Down Syndrome, Barcelona, Spain

## Abstract

Down Syndrome and Alzheimer's Disease: A New Paradigm in AD Research

Adults with Down syndrome (DS) are the largest genetically defined population at risk for Alzheimer's disease (AD), due to lifelong overexpression of the *APP* gene on chromosome 21. As a result, virtually all individuals with DS develop AD neuropathology by mid‐adulthood, making Down syndrome‐associated Alzheimer's disease (DSAD) one of the most striking examples of genetically determined AD.

In recent years, DSAD has emerged from the margins to become a central focus in Alzheimer's research. Large‐scale international consortia, such as ABC‐DS and Horizon21, have transformed our understanding of its natural history and biology, leading to DSAD's inclusion in the newly proposed Alzheimer's Association diagnostic criteria. Importantly, insights from DSAD have also laid the groundwork for a proposal to reconceptualize APOE4 homozygotes as part of the broader family of genetically determined AD.

These advances have been accompanied by unprecedented increases in funding and scientific attention, positioning DSAD as a powerful model for early detection, biomarker development, and disease modification. This plenary will highlight the extraordinary progress in the field, from population‐based studies to fluid and imaging biomarkers, and the emergence of clinical trials that mark a new era in AD prevention and treatment for this high‐risk population.

